# Triggering word learning in children with Language Impairment: the effect of phonotactic probability and neighbourhood density*

**DOI:** 10.1017/S0305000913000445

**Published:** 2013-11-06

**Authors:** CRISTINA MCKEAN, CAROLYN LETTS, DAVID HOWARD

**Affiliations:** Newcastle University

## Abstract

The effect of phonotactic probability (PP) and neighbourhood density (ND) on triggering word learning was examined in children with Language Impairment (3;04–6;09) and compared to Typically Developing children. Nonwords, varying PP and ND orthogonally, were presented in a story context and their learning tested using a referent identification task. Group comparisons with receptive vocabulary as a covariate found no group differences in overall scores or in the influence of PP or ND. Therefore, there was no evidence of atypical lexical or phonological processing. ‘Convergent’ PP/ND (High PP/High ND; Low PP/Low ND) was optimal for word learning in both groups. This bias interacted with vocabulary knowledge. ‘Divergent’ PP/ND word scores (High PP/Low ND; Low PP/High ND) were positively correlated with vocabulary so the ‘divergence disadvantage’ reduced as vocabulary knowledge grew; an interaction hypothesized to represent developmental changes in lexical–phonological processing linked to the emergence of phonological representations.

## INTRODUCTION

Many children with Language Impairment (LI)^1^ have difficulty learning new words (Alt & Plante, 2006; Alt, Plante & Creusere, 2004; Gray, 2003, 2004, 2005; Gray & Brinkley, 2011) and these difficulties extend across childhood. As toddlers, most children with LI are slow to begin the process of lexical acquisition, often presenting as ‘late talkers’ (Reilly *et al*., 2009); as primary school children they require many more exposures than Typically Developing (TD) children to learn a word (Gray, 2003, 2004) and create under-specified semantic representations (McGregor, Newman, Reilly & Capone, 2002); and, as adolescents, their vocabulary test scores fall further and further behind their TD peers (Stothard, Snowling, Bishop, Chipchase & Kaplan, 1998). Gaining a greater understanding of the nature and causes of these word learning difficulties could bring insights with benefits both to practice and theory relating to children with LI.

Poor vocabulary and word learning abilities have significant implications for children's academic outcomes. Vocabulary knowledge is closely linked to the development of literacy skills such as phonological awareness, decoding, and reading comprehension abilities (Biemiller, 2003; Biemiller & Slonim, 2001; Van Weerdenburg, Verhoeven, Bosman & van Balkom, 2011), and to the advanced discourse skills, such as narration and exposition, required for success in academic examinations and in the modern workplace (Nippold, 1998). Supporting vocabulary development in children with LI could therefore reduce the negative sequelae in educational outcome often associated with this disorder (Snowling, Adams, Bishop & Stothard, 2001).

In a number of explanatory models of LI, a phonological processing impairment is seen as core to the ontogeny of the disorder (Bishop, 2000; Bowey, 2006; Chiat, 2001; Maillart, Schelstraete & Hupet, 2004; Mainela-Arnold & Evans, 2005; Seiger-Gardner & Brooks, 2008). Furthermore, impairments in the nature of lexical knowledge/representations have also been reported (Edwards & Lahey, 1999; McGregor *et al*., 2002; Seiger-Gardner & Brooks, 2008). Recent models of phonological development underline the mutually dependent and interactive relationship between phonological knowledge and lexical knowledge over development (Beckman & Edwards, 2000; Edwards, Munson & Beckman, 2011; Garlock, Walley & Metsala, 2001; Gupta & Tisdale, 2009; Pierrehumbert, 2003; Walley, Metsala & Garlock, 2003; Werker & Curtin, 2005). That is, the nature of phonological representations are thought to be dependent upon the nature of lexical knowledge and, conversely, lexical learning is thought to be dependent on the nature of phonological representations, these representations affecting the nature, efficiency, and capacity of a child's phonological processing and thence, word learning. If phonological processing is crucial to the ontogeny of LI, and the development of the phonological knowledge which underpins processing is inextricably linked to lexical knowledge, then increasing our understanding of how children with LI learn words could provide key insights as to the nature of this ‘core deficit’.

This study seeks to better understand the process of word learning in children with LI and, specifically, to explore whether the influence of phonological and lexical variables on the process of ‘triggering’ word learning in this group of children differs from those of TD children. By determining whether and how lexical and phonological processing in LI differs from TD children during word learning, new insights could be gained to inform interventions to promote faster and more robust word learning in children with LI.

### Word learning in TD children

Learning a new word is a highly complex achievement involving a large number of constraints and biases which influence the child (Golinkoff *et al*., 2000); a number of stages in the process of learning a particular word (from initial mapping to a fully developed representation) (Hoover, Storkel & Hogan, 2010); and changes across development in the processes brought to bear when learning a word (Hirsh-Pasek, Golinkoff & Hollich, 2000; McKean, Letts & Howard, 2013; Storkel, 2009). Hoover and colleagues (2010) describe the process of word learning as consisting of three stages: triggering, configuration, and engagement. Triggering refers to the process of recognizing when a new word has been encountered such that the child or adult then ‘switches on’ the learning process (McKean *et al*., 2013; Storkel, Armbruster & Hogan, 2006). This triggering then leads to configuration during which a new lexical representation is created. Over time, engagement then occurs, wherein links between the novel lexical item and existing lexical knowledge are made at lexical, semantic, and phonological levels of representation, hence building similarity neighbourhoods and connections within the lexicon (Hoover *et al*., 2010; Leach & Samuel, 2007; Storkel *et al*., 2006; Storkel & Lee, 2011).

In recent research, two form characteristics of novel words have been shown to influence word learning in TD children: Phonotactic Probability (PP), which is a measure of the likelihood frequency of particular sequences of sounds in a language (Vitevitch & Luce, 1999); and Neighbourhood Density (ND), which is a measure of the numbers of lexical neighbours in the similarity neighbourhood of a word (Vitevitch & Luce, 1999) and is usually calculated as the number of words differing from the target by one phoneme substitution, addition, or deletion (cf. Goldrick, Folk & Rapp, 2010, for alternative metrics for ND). It is thought that these variables exert influence at different levels of speech processing: PP at the phonological level of representation and ND at the lexical level (Vitevitch & Luce, 1998, 1999). It is therefore possible, by manipulating these variables in experimental tasks, to examine the nature of processing at each level and the nature of interactions between those levels (cf. Werker & Curtin, 2005, for a discussion of an additional ‘phonetic-indexical’ level of processing to consider in word learning).

PP and ND are highly correlated (Vitevitch & Luce, 1998) and so early research into their influence on word learning tended to covary these factors (Storkel, 2001, 2003; Storkel & Hoover, 2006; Storkel & Rogers, 2000), demonstrating that children between the ages of three and thirteen years learn words with high PP/high ND more readily than those with low PP/low ND (Storkel, 2001, 2003; Storkel & Rogers, 2000). It is only very recently that the independent influences of PP and ND on word learning in children have been explored (Hoover *et al*., 2010; McKean *et al*., 2013; Storkel, 2009; Storkel & Lee, 2011; Storkel, Maekawa & Hoover, 2010). These studies have demonstrated not only that PP and ND exert separable influences on word learning in children, but also that these influences vary across the stages of the word learning process (Hoover *et al*., 2010; Storkel & Lee, 2011) and across development (Maekawa & Storkel, 2006; McKean *et al*., 2013; Storkel, 2009).

As identified above, this study seeks to better understand the process of word learning in children with LI and, specifically, to explore whether the influence of phonological (PP) and lexical (ND) variables on the process of ‘triggering’ word learning in this group of children differs from those of TD children. The following therefore summarizes current research relating to this issue, first with respect to TD children, and second, those with LI.

#### The influence of PP and ND on triggering in TD children

The separable effects of PP and ND on triggering have been investigated in a small number of studies through the examination of preschool children's immediate retention of novel words heard in a story context (Hoover *et al*., 2010; McKean *et al*., 2013; Storkel & Lee, 2011). Both Hoover *et al*. (2010) (in children aged between 3;00 and 5;10) and Storkel and Lee (2011) (in children aged between 4;00 and 4;11) found that the optimal word form characteristics for triggering word learning were low PP and low ND. Hence, it would appear that it easier to identify that a word is novel rather than known where the child knows few similar words with which it could be confused, and where the phonological form is relatively infrequent and therefore noticeably ‘odd’ or ‘new’ (Storkel *et al*., 2006). The findings of McKean *et al*. (2013) generally concur, identifying a low ND advantage (in children aged between 3;01 and 5;02) but also identifying developmental change in the influence of PP from a high PP advantage to a low PP advantage over the age range studied.

In addition to a Low PP/Low ND advantage, Hoover *et al*. (2010) identify a ‘convergence advantage’ such that High PP/High ND and Low PP/Low ND words (i.e., convergent PP/ND characteristics) were easier to learn than either Low PP/High ND or High PP/Low ND (i.e., divergent PP/ND characteristics). This significant interaction between PP and ND is not found in adults (Storkel *et al*., 2006) and so developmental change in the nature of these biases would seem to exist. However, when and how this change occurs has yet to be empirically determined.

Research is therefore beginning to uncover the role which PP and ND play in triggering word learning in TD children. Much less is known, however, about the influence of ND and PP on word learning generally, and triggering specifically, in children with LI.

#### The influence of PP and ND on triggering in children with LI

To date, three studies have explored the influence of PP on word learning in preschool children with LI (Alt & Plante, 2006; Gray & Brinkley, 2011; Gray, Brinkley & Svetina, 2012). The influence of ND, however, has not been explicitly tested, rather it has either been considered post-hoc (Alt & Plante, 2006), or has been held constant at zero (Gray & Brinkley, 2011; Gray *et al*., 2012).

In all three studies, probes of lexical learning (e.g., referent identification, recognition, and production) demonstrated no significant effect of PP on triggering either for preschool TD children or for children with LI (aged 3;01–5;11). As neither group were sensitive to PP effects, and yet TD children's triggering of word learning has been shown to be affected by PP in other studies (Hoover *et al*., 2010; McKean *et al*., 2013; Storkel & Lee, 2011), these null results must be interpreted with caution. As Gray and colleagues acknowledge (Gray & Brinkley, 2011; Gray *et al*., 2012), the chosen stimuli in their studies, which varied in PP only with respect to embedded, two-phoneme sequences, could have exerted a smaller effect than stimuli used in other studies, which varied in PP for all phonemes and phoneme sequences (Hoover *et al*., 2010; McKean *et al*., 2013; Storkel & Lee, 2011). Scores for triggering tend to be low and so the combination of low scores and a smaller PP effect could have produced results with insufficient power to discern any influence of PP on triggering. Similarly, the recognition task used by Alt and Plante (2006) to probe lexical learning produced very low scores, which again could mask an effect of PP. It is therefore possible that PP does influence triggering in children with LI but, to date, studies tapping lexical learning have not had sufficient power to detect this influence.

It should be noted that Alt and Plante (2006) did find a significant effect of PP on the process of semantic mapping, a task yielding higher scores than were found for lexical mapping, such that the children with LI were able to map as many semantic features as the TD group where the novel word had a high PP, but mapped significantly fewer semantic features than the TD group in the low PP condition. With its focus on semantic mapping it is, perhaps, moot whether this study's findings speak to the process of triggering or whether they may be better conceptualized as part of configuration.

It is therefore not clear whether PP affects lexical learning during triggering in children with LI, and the independent effects of both PP and ND on word learning have never been explored in this group of children. This study therefore seeks to determine the separable and interactive effects of PP and ND on triggering, the earliest stage of word learning, in children with LI, and to compare this to TD children. In order to achieve this aim, stimuli varying orthogonally in PP and ND are required, and must be presented in an experimental task which taps the earliest stage of word learning, yet produces sufficiently high scores to detect any effects of PP. To that end we replicated McKean *et al*.'s (2013) study with a group of children with LI. This experimental paradigm includes stimuli varying orthogonally in PP and ND and has been shown to be able to detect differences in the influence of PP and ND at immediate retention and after minimal exposures through the analysis of children's correct responses in a referent identification task (McKean *et al*., 2013).

As previously described, lexical learning is thought to be dependent on the nature of phonological representations, such that these representations affect the nature, efficiency, and capacity of a child's phonological processing, and thence their word learning. In a reciprocal fashion, the nature of phonological representations is thought to be dependent upon the nature of lexical knowledge (Beckman & Edwards, 2000; Edwards *et al*., 2011; Garlock *et al*., 2001; Gupta & Tisdale, 2009; Pierrehumbert, 2003; Walley *et al*., 2003; Werker & Curtin, 2005). It is therefore possible that any differences which might be found between children with LI and TD children in the influence of lexical variables (as indexed by ND) and phonological variables (as indexed by PP) could result purely from differences in the children's levels of vocabulary knowledge, representing a delayed pattern of processing which is in line with the child's lexical knowledge, rather than an atypical processing bias. This study therefore compares preschool children with LI to TD children with respect to their vocabulary knowledge. In this way, we aim to determine whether atypical lexical or phonological processing exists in children with LI during triggering of word learning, over and above that which can be explained by their level of vocabulary development. In this way we aim to uncover whether a ‘core deficit’ in phonological processing is influencing triggering of word learning in LI (Bishop, 2000; Bowey, 2006; Chiat, 2001; Maillart *et al*., 2004; Mainela-Arnold & Evans, 2005; Seiger-Gardner & Brooks, 2008).

This study therefore asks the following research questions:

1. How do PP and ND influence the triggering of word learning in children with LI?
2. Do the effects of PP and ND interact during the triggering of word learning in children with LI?
3. Do the effects of PP and ND differ from those found for TD children when compared with respect to vocabulary knowledge, hence indicating atypicality in lexical and/or phonological processing over and above that which can be explained by lexical knowledge?

## METHOD

### Participants

Ethical approval was obtained to recruit TD children and those with LI from schools and Speech and Language Therapy Services in the north of England. A total of 52 participants were recruited to the study. Thirteen children aged between 3;04 and 6;09 with LI were recruited from Speech and Language Therapist's caseloads (Omnibus Language test score [either comprehension, expression, or both] falling ⩽1·25 *SD* below the mean or ⩽10th centile; a non-verbal test score ⩾1 *SD* below the mean or ⩾ the 16th centile; excluding any child with a hearing impairment, visual impairment, physical disability, autism diagnosis, motor-speech difficulty, and those children who spoke English as an additional language). One child with LI (aged 3;08) refused to complete some sections of the word learning task and so was excluded from the analysis, resulting in 12 participants in the LI group. The TD children were recruited from a Foundation Stage Unit of a local primary school (providing education to children aged 3;00–5; 11). The TD participants were 38 monolingual English speakers in the age range 3;01–5;02 with no identified developmental or language difficulties. The language, non-verbal skills, and hearing status of both groups of children were assessed using the Reynell Developmental Language Scales III (RDLS III: Edwards *et al*., 1997), the British Ability Scales Block Building Subtest (BAS: Elliot, Smith & McCulloch, 1996), and the ASHA Audiologic Screening Protocol (ASHA, 1997), respectively. The number of participants in each age group is presented in the ‘Appendix’ and summary participant data in Table 1.

**Table 1.** Participant summary characteristics

Group	N	Age (months)	RDLS III Comprehension centile scores	RDLS III Expression centile scores	BAS Block Building centile scores	EOWPVT Raw scores^a^	ROWPVT Raw scores^a^
TD	38	51·68 (7·33)	65·92** (20·50)	46·32** (21·09)	44·5 (26·22)	48·79** (10·73)	51·30* (9·35)
LI	12	55·42 (15·26)	6·58** (10·31)	1·58** (1·16)	43·92 (28·93)	36·50** (12·40)	43·58* (12·82)

note: RDLS III = Reynell Developmental Language Scales III (Edwards *et al*., 1997); BAS = British Ability Scales (Elliot *et al*., 1996); EOWPVT = Expressive One Word Picture Vocabulary Test (Brownell, 2000a); ROWPVT = Receptive One Word Picture Vocabulary Test (Brownell, 2000b).

a Raw scores are provided for the vocabulary tests rather than centile scores to enable a clearer comparison as to the degree to which the scores of the two groups overlap despite some differences in age.

* *p* < ·05, two-tailed t tests; ** *p* < ·01, two-tailed tests.

### General procedures

The TD children were seen in a quiet area of their school and the children with LI either in their school or at home, depending on parental preference. The length of session was tailored to the child's individual level of attention and motivation, and so assessment for inclusion and the experimental data collection for this study were completed over two to four sessions with each child, each session lasting between 20 and 45 minutes. The word learning task and measures of word learning were completed in one session.

### The word learning task

The fast mapping task presented in Storkel (2001) was adapted to include stimuli orthogonally varying ND and PP. As the children in this study were likely to have wide-ranging language abilities, the sentence structures used in the story were simplified and the semantic categories of the referent objects changed to those of earlier developing superordinate categories than in the original task (toys, pets, food, and vehicles as compared to toys, pets, horns, and candy machine; Storkel, 2001). In this way, the potential confound of the effect of semantic and syntactic receptive language abilities on word learning performance was minimized.

The children were introduced to the nonword–referent pairings in a story involving two aliens (Jim and Bob) going shopping. At each new location the aliens each bought or used a new referent from one of the categories; buying a toy each at the toy shop, a pet each at the pet shop, eating some food at the café, and catching a rocket home at the ‘rocket stop’. During the story each word–referent pairing was presented six times. To increase the number of repetitions while maintaining the child's attention, a ‘storyboard activity’ was also completed. This provided the opportunity for two additional repetitions of the word–referent pairings. The storyboard activity involved the children re-enacting the story by moving small figures of Jim and Bob along a board with a street depicted on it. The children therefore heard a total of eight repetitions of each novel word over the whole task. The complete story and storyboard script can be found in McKean *et al*. (2013). The story structure and sentence structures were designed to be as simple as possible. The novel word was presented in sentence-final position and the carrier sentences contained a maximum of three clause elements. The carrier sentences used were: ‘I want a X’; ‘Here is the X’; ‘Jim/Bob has the X’; ‘This one is the X’; ’ I like the X’; ‘Jim/Bob bought/ate/caught the X’. The protocol was administered ‘live voice’ as, during piloting, this was found to be the most successful method for maintaining the attention of the youngest children. The first author administered the tasks for all of the study participants.

#### The nonwords

A range of CVC nonwords were created using those consonants which Grunwell (1985) describes as occurring in the speech of children aged 3;06–4;06 (/m n p b t d k g s f w j h, and ʃ/). The Phonotactic Probability and Neighbourhood Density of the candidate nonwords were calculated and stimuli chosen varying PP and ND orthogonally with two exemplars of each of the four subcategories in the story (toys, pets, food, vehicles) (Table 2). The PP and ND of the candidate list of nonwords were calculated; PP was calculated from data in the CELEX database (Baayen, Piepenbrock & Gulikers, 1995) and validated using Vitevitch's online ‘Phonotactic Probability Calculator’ (Vitevitch & Luce, 2004) (Table 2); Neighbourhood Density was defined as the number of neighbours differing from the target by one phoneme substitution, addition, or deletion and was also calculated using the CELEX database (Baayen *et al*., 1995).

**Table 2.** Form and referent characteristics of the chosen stimuli

	Word	ND*	Log normalized	Positional segment	Biphone	Referent	Referent
			PP^a^	frequency^b^	frequency^b^	Category	
High ND	teɪn	34	0·6232	·1698	·0042	toy	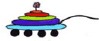
High PP	bɑɪn	38	1·0597	·1816	·0071	pet	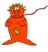
Low ND	hɔɪf	4	−1·6534	·0625	·0042	toy	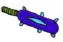
Low PP	jɔʃ	7	−1·607	·0321	·0006	pet	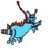
High ND	heɪm	25	−0·6206	·1180	·0026	food	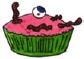
Low PP	ʃeɪt	29	−0·0386	·1049	·0037	vehicle	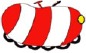
Low ND	hɑn	9	0·7673	·1960	·0156	vehicle	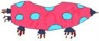
High PP	ɡek	13	1·0869	·1524	·0076	food	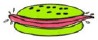

notes: ^a^ CELEX; ^b^ Hoosier Mental lexicon.

An estimate of ‘high’ and ‘low’ neighbourhood density for young children was determined by considering the characteristics of the three-phoneme words in the Morrison, Chappell, and Ellis word list (Morrison, Chappell & Ellis, 1997) with an age of acquisition of less than five years, and the three-phoneme nouns in the British English version of the CDI (Fenson *et al*., 1997). The mean and standard deviation of the ND for these words was calculated (*μ* = 23·7; *SD* = 8). As a result, we operationally defined High ND as ⩾ the mean and Low ND ⩽1 *SD* below the mean. These criteria ensured that the chosen stimuli were substantially different from one another in ND whilst also producing sufficient numbers of candidate stimuli. The High ND/High PP and Low ND/Low PP nonwords candidate stimuli were selected with the highest and lowest values respectively for each measure. For the Low ND/High PP and High ND/Low PP stimuli, those nonwords which fulfilled the ND criteria for high and low status described above were identified and then, from those subsets of candidate stimuli, those with the lowest or highest possible PP values chosen. Stimuli were chosen to maximize the number of different phonemes used across the nonwords in order to make the words maximally distinct. The final stimuli were chosen to contain the fewest possible overlapping phonemes whilst containing only the consonants /m n p b t d k g s f w j h, and ʃ/ and varying orthogonally PP and ND.

Two-sample *t*-tests demonstrated that the differences between the stimuli categorized as ‘high’ and ‘low’ PP and ND were statistically significant (ND: *t*(6) = 6·81, *p* < ·001 (one-tailed); PP log normalized: *t*(3·49) = 4·55, *p* = ·007 (one tailed); PP positional segment frequency: *t*(6) = 4·39, *p* = ·002 (one-tailed); PP biphone frequency: *t*(6) = 2·28, *p* = 0·032 (one-tailed)).

#### The referents

The eight resulting novel nonwords were each paired with a novel referent in a story context. The story was set on an alien planet and the novel objects represented items from the categories toys, food, pets, and vehicles with two items in each semantic category (Table 2). The semantic categories were chosen from those which appear in the CDI and so exist in the lexicon of typically developing children aged 2;06 (Fenson *et al*., 1997). Novel words were assigned to the referents so that for each semantic category pair (toys, foods, pets, and vehicles) the novel word pair contrasted high and low PP and high and low ND. To ensure that there were no significant differences in PP and ND characteristics between the categories, a series of one-way ANOVAs were completed. There was no main effect of semantic category for any of the ND or PP metrics (ND: *F*(3,4) = 0·02, *p* = ·995, partial *η*^2^ = 0·02; PP log normalized: *F*(3,4) = 0·17, *p* = ·91, partial *η*^2^ = 0·12; PP positional segment frequency: *F*(3,4) = 0·14, *p* = ·93, partial *η*^2^ = 0·10; PP biphone frequency: *F*(3,4) = 0·55, *p* = ·67, partial *η*^2^ = 0·29). The semantic categories and referents were not counterbalanced across participants. To check whether any individual item contributed especially to the results, the untransformed residuals from the ANCOVA, collapsed across groups, were standardized, and tested for homogeneity: the result was not significant (*χ*^2^(7) = 0·078, *p* > ·05).

#### The measures of learning

The children's knowledge of the newly learned words was assessed during and immediately after the word learning task, using a comprehension probe. This consisted of the child finding the correct referent from a choice of four pictures (‘Show me the X’). The probe was presented at three points in the experiment: for each new category pair after the story episode which introduced them (i.e., the toys were tested after the toy shop visit, the pets after the pet shop visit, and so on); for all eight novel words at the end of the story; and again, for all eight words at the end of the story board game.

The comprehension probe tested the abilities of the children to fast-map a representation of the phonological string for the novel word (a lexical representation), a representation of the referent for the novel word (a semantic representation), and a link between the two representations. The child was asked to ‘Show me the X’, and selected the corresponding picture from a choice of four: (1) the Target referent; (2) a Related Distracter (the referent from the same semantic category in the story); (3) an Unrelated Distracter (another referent from the story from a different semantic group); (4) a Foil (a novel object which did not appear in the story).^2^

#### Scoring

Responses on the comprehension probe were recorded by circling the child's response on a score sheet. Totals for each response (Target, Related Distracter, Unrelated Distracter, and Foil) were tallied across the three assessment points. There was no significant difference in scores at each time-point and so scores were collapsed across time-points. Children's responses were scored as correct (choosing the Target), semantic error (choosing the Related Distracter), or unrelated error (choosing either the Unrelated Distracter or the Foil). The dependent variable total correct responses was analyzed to explore word learning ability.

### Vocabulary knowledge

The children were tested using the Expressive One Word Picture Vocabulary Test (EOWPVT) (Brownell, 2000a) and the Receptive One Word Picture Vocabulary Test (ROWPVT) (Brownell, 2000b). ROWPVT raw scores were preferred as the covariate for this study for two reasons. First, this task more closely aligns to the measure of word learning in the current study (i.e., referent identification). Second, as many children with SLI have Word Finding Difficulties in addition to broader language difficulties (i.e., a significant and atypical discrepancy between their receptive and expressive vocabulary abilities; Dockrell & Messer, 2007; Kail & Leonard, 1986; Leonard, Nippold Kail & Hale, 1983), it is possible that an expressive vocabulary score may be a less ‘pure’ measure of lexical knowledge than a receptive vocabulary score for this group of children. That is, errors would capture both a lack of knowledge and a lack of ability to retrieve that knowledge for naming, therefore adding additional ‘noise’ to this variable and producing a less reliable estimate of the child's level of vocabulary knowledge than a receptive vocabulary score.

## RESULTS

**Fig. 1.**
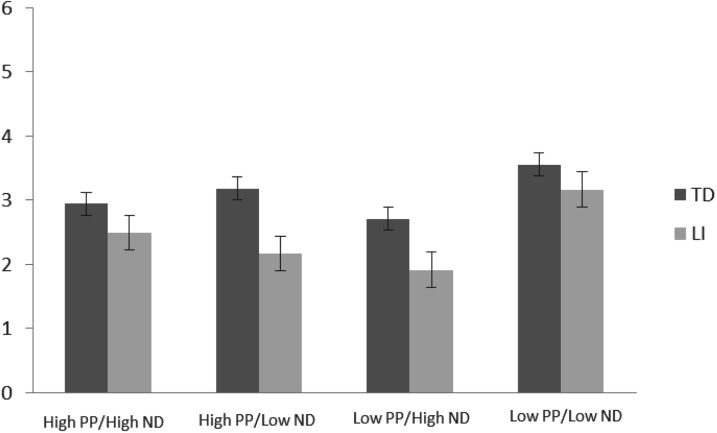
Mean scores for each PP/ND combination for children with Language Impairment (LI) and Typical Development (TD). The maximum possible score for each PP/ND combination was 6. Standard errors are represented by error bars.

In order to determine the separate and interactive effects of PP and ND in triggering word learning in children with LI, and to compare these effects to TD children a 2*2*2 mixed ANCOVA analysis with group (LI and TD) as the between-subjects factor, and PP (High and Low) and ND (High and Low) as the within-subjects factors, and with receptive vocabulary score as the covariate, was completed. Levene's test demonstrated that the assumption of homogeneity of variances had been violated. The dependent variable was therefore log-transformed to enable ANCOVA analyses to be applied. All results reported below relate, therefore, to the log-transformed data. All figures represent the untransformed data for ease of interpretation. Significant main effects and interactions were followed by planned ANCOVA contrasts. Mean scores for each PP/ND combination for each group are presented in Figure 1 and for the individual children with LI in Table 3.

**Table 3.** Total correct responses in referent identification task of children with LI

		Number correct				
Participant	Age	High PP High ND	High PP Low ND	Low PP High ND	Low PP Low ND	Total
1	3;07	2	3	1	3	**9**
2	3;04	3	3	2	2	**10**
3	3;04	3	2	1	3	**9**
4	3;06	1	1	1	3	**6**
5	3;11	2	0	0	6	**8**
6	4;07	2	0	2	2	**6**
7	4;04	2	4	3	3	**12**
8	4;02	1	0	2	4	**7**
9	5;05	4	3	3	4	**14**
10	6;11	5	4	3	3	**15**
11	6;06	4	2	2	4	**12**
12	6;02	1	4	3	1	**9**
**Total**		**30**	**26**	**23**	**38**	

note: A maximum score of 6 was possible for each word form condition, and the maximum possible total score was 24.

### Group comparisons

The ANCOVA analyses described above revealed a significant main effect of Vocabulary (*F*(1,46) = 4·18; *p* = ·047; partial *η*^2^ = 0·08), but no other significant main effects (Group: *F*(1,46) = 0·38; *p* = ·54; partial *η*^2^ = 0·01; PP: *F*(1,46) = 0·78; *p* = ·38; partial *η*^2^ = 0·02; ND: *F*(1,46) = 1·62; *p* = ·21; partial *η*^2^ = 0·03). The main effect of Vocabulary was qualified by a significant three-way interaction between ND, PP, and Vocabulary (*F*(1,46) = 11·31; *p* < ·01; partial *η*^2^ = 0·20), and a significant ND × PP interaction (*F*(1,46) = 15·80; *p* < ·01; partial *η*^2^ = 0·26). No other significant effects were present (PP x Group: *F*(1,46) = 2·52; *p* = ·12; partial *η*^2^ = 0·05; PP × Vocabulary: *F*(1,46) = 0·61; *p* = ·44; partial *η*^2^ = 0·01; PP × Group × Vocabulary: *F*(1,46) = 2·27; *p* = ·14; partial *η*^2^ = 0·05; ND × Group: *F*(1,46) = 0·15; *p* = ·70; partial *η*^2^ = 0·003; ND × Group × Vocabulary: *F*(1,46) = 0·28; *p* = ·60; partial *η*^2^ = 0·006; PP × ND × Group: *F*(1,46) = 1·04; *p* < ·01; partial *η*^2^ = 0·26; PP × ND × Group × Vocabulary: *F*(1,46) = 0·28; *p* = ·31; partial *η*^2^ = 0·02). Therefore there were no significant differences between the children with LI and the TD children overall or in the nature of the effects of PP and ND.

### The interactive effects of ND and PP

The significant ND × PP and ND × PP × Vocabulary interactions were explored using a series of repeated measures ANCOVAs exploring the effect of PP at different levels of ND and vice versa.

#### The effect of PP at different levels of ND

**Fig. 2.**
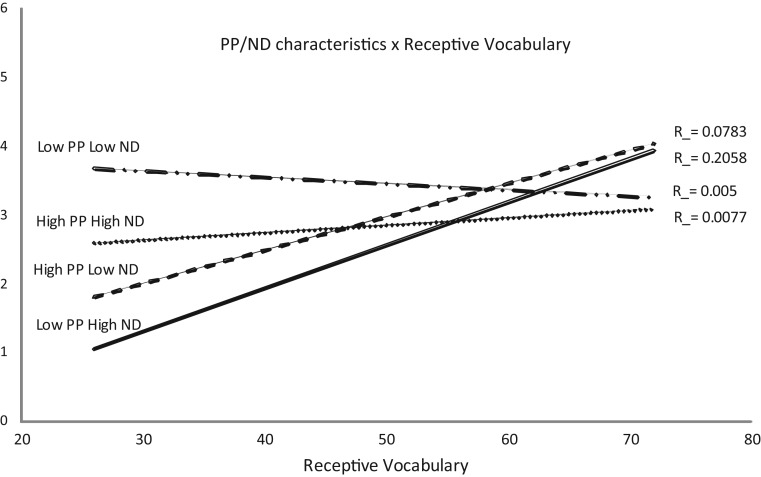
Referent identification scores for each PP/ND combination collapsed across groups, and their relationship with receptive vocabulary scores.

For High ND words a repeated measures ANCOVA found a significant main effect of PP (such that high PP words were advantaged) (*F*(1,48) = 6·49; *p* = ·01; partial *η*^2^ = 0·11), and a significant interaction between Vocabulary and PP (*F*(1,48) = 4·99; *p* = ·03; partial *η*^2^ = 0·09). This Vocabulary × PP interaction was explored using a correlational analysis. Words with Low PP/High ND were positively correlated with Vocabulary (Pearson's *r* = 0·41, *p* < ·01) but those with High PP/High ND were not (Pearson's *r* = 0·10, *p* = ·50) (see Figure 2). Hence, children found Low PP/High ND words (i.e., ‘divergent’ PP/ND characteristics) more difficult to learn than High PP/High ND words (i.e., ‘convergent’ PP/ND characteristics). However, the significant positive correlation between vocabulary and Low PP/High ND word learning suggests that this ‘divergence disadvantage’ decreased as vocabulary knowledge grew.

For Low ND words, a significant main effect of PP was present (such that low PP words were advantaged: *F*(1,48) = 10·60; *p* < ·01; partial *η*^2^ = 0·18), and a significant PP × Vocabulary interaction (*F*(1,48) = 7·72; *p* < ·01; partial *η*^2^ = 0·14). Correlational analysis revealed that High PP/Low ND words were correlated with vocabulary (Pearson's *r* = 0·34, *p* = ·02), but Low PP/Low ND words were not (Pearson's *r* = −0·10, *p* = ·51) (see Figure 2). Hence, children found High PP/Low ND words (i.e., ‘divergent’ PP/ND characteristics) more difficult to learn than Low PP/Low ND words (i.e., ‘convergent’ PP/ND characteristics). However, the significant positive correlation between vocabulary and High PP/Low ND word learning suggests that this ‘divergence disadvantage’ decreased as vocabulary knowledge grew.

#### The effect of ND at different levels of PP

For High PP words, the effect of ND was explored using a repeated measure ANCOVA, which found a significant main effect of ND (such that High ND was advantaged: *F*(1,48) = 4·63; *p* = ·04; partial *η*^2^ = 0·09), and a significant ND × Vocabulary interaction (*F*(1,48) = 4·23; *p* = ·045; partial *η*^2^ = 0·08), reflecting the significant correlation between High PP/Low ND words and vocabulary which was not present for High PP/High ND words (see above). Hence, children found High PP/Low ND words (i.e., ‘divergent’ PP/ND characteristics) more difficult to learn than High PP/High ND words (i.e., ‘convergent’ PP/ND characteristics). However, the significant positive correlation between vocabulary and High PP/Low ND word learning suggests that this ‘divergence disadvantage’ decreased as vocabulary knowledge grew.

For Low PP words, the repeated measures ANCOVA found a significant main effect of ND such that Low ND words were advantaged (*F*(1,48) = 13·39; *p* < ·01; partial *η*^2^ = 0·220, and a significant ND × Vocabulary interaction (*F*(1,48) = 8·80; *p* = ·01; partial *η*^2^ = 0·160, reflecting the significant correlation between Low PP/High ND words and vocabulary which was not present for Low PP/Low ND words, reported above. Hence, children found Low PP/High ND words (i.e., ‘divergent’ PP/ND characteristics) more difficult to learn than Low PP/Low ND words (i.e., ‘convergent’ PP/ND characteristics). However, the significant positive correlation between vocabulary and Low PP/High ND word learning suggests that this ‘divergence disadvantage’ decreased as vocabulary knowledge grew.

In summary, there were no significant differences between the children with LI and the TD children in their overall ability to learn words when compared with respect to vocabulary knowledge. Furthermore, the nature of the effects of PP and ND did not differ between groups. PP and ND interacted such that there was a significant ‘divergence disadvantage’ or ‘convergence advantage’ (i.e., High PP/High ND words were easier to learn than High PP/Low ND or Low PP/High ND, and Low PP/Low ND words were easier to learn than Low PP/High ND and High PP/Low ND words). Significant positive correlations between vocabulary and scores for High PP/Low ND and Low PP/High ND words demonstrated that this ‘divergence disadvantage’ decreased as vocabulary knowledge grew.

## DISCUSSION

The current study was designed to explore whether young children with LI differ from TD children in the nature of the effects of lexical (ND) and phonological (PP) variables on the process of triggering word learning, both independently and interactively. Comparisons were made with vocabulary as a covariate to consider whether any identified differences could be explained as resulting from differences in the children's levels of vocabulary knowledge, hence representing a delayed pattern of processing rather than an atypical processing bias. The following discusses, in turn, the findings with respect to triggering word learning in children with LI and those with respect to the nature of interactivity between ND and PP.

### Word learning in children with LI

This study found no overall group differences in the abilities of the children to learn new words at the first stage of word learning (triggering) when groups were compared with respect to vocabulary knowledge, and no significant interactions between group and PP or group and ND, suggesting that children with LI are influenced by these lexical and phonological characteristics in a similar way to TD children. These results therefore do not support the hypothesis that phonological processing is a core deficit in LI (Bishop, 2000; Bowey, 2006; Chiat, 2001; Maillart *et al*., 2004; Mainela-Arnold & Evans, 2005; Seiger-Gardner & Brooks, 2008); rather, they suggest that the delay found in phonological processing in children with LI may result from, rather than cause, the more limited lexical knowledge of children with LI, and therefore that the cause of the deficit in word learning is yet to be established. Such an assumption may be premature as it is possible that a phonological processing deficit may exert an effect on the later stages of word learning (i.e., configuration and engagement). However, Gray and colleagues (Gray & Brinkley, 2011; Gray *et al*., 2012) found no difference in the effect of PP between TD and LI groups even for these later stages of word learning.

On the whole, therefore, the results of the current study align with those of Gray and colleagues (Gray & Brinkley, 2011; Gray *et al*., 2012) in finding no differences between children with LI and their TD peers in word learning ability at the earliest stage of the word learning process, and no difference in the effect of PP. Further, our findings demonstrate for the first time that the effects of ND on triggering word learning do not differ between TD children and those with LI.

Where, then, is the source of the word learning difficulties found in children with LI? A recent meta-analysis of studies of word learning in LI identified a number of issues of relevance to this question and to the interpretation of the current study's findings (Kan & Windsor, 2010). First, they demonstrated that children with LI are significantly poorer than their age-matched peers in word learning tasks, but are not poorer than their language-matched peers; second, that the differences between LI and TD groups were larger in studies where children had high exposures to the novel words (i.e., studies of consolidation rather than triggering); and third, that both age and cognition contribute to the size of the group differences observed. That is, group differences were more often seen for preschool children with LI than school-aged children, and cognition was found to be a significant predictor of word learning ability in a moderator analysis.

Kan and Windsor's (2010) findings offer support to the validity of the current study's null result, suggesting that the absence of group differences reported here do not simply reflect insufficient power due to the small number in the LI group. That is, their findings, from a large, powerful dataset, that no differences exist between language-matched groups and children with LI and that group differences are small at the earliest stage of word learning (triggering), support the conclusion that difficulties in triggering word learning are unlikely to be the source of the significant word learning difficulties found in children with LI.

It therefore seems logical to suggest that the word learning difficulties found in children with LI occur during the later stages of the word learning process (engagement and/or configuration); a suggestion which could be supported by Alt and Plante's (2006) finding, in a task which may be tapping the early stages of configuration, that children with LI mapped significantly fewer semantic features than their TD peers when the novel word had low PP. However, Gray and colleagues (Gray & Brinkley, 2011; Gray *et al*., 2012) also explored the later stages of word learning in LI and found no group differences when compared to either age-matched or language-matched TD peers. Kan and Windsor's (2010) finding, that both age and cognition contribute to the size of the group differences observed in word learning studies, may hold the key to these differences between studies, pointing to more general processing and cognitive abilities as potentially playing an important role in word learning for children with LI. It is likely that the word learning contexts used both in the current study and in Gray and colleagues (Gray & Brinkley, 2011; Gray *et al*., 2012) do not reflect the level of processing demands which learning ‘in the wild’ present to the child, and hence provide sufficient levels of support for the child with LI to achieve typical levels of word learning. For example, in this study and those of Gray and colleagues, the target referents are clearly identified, removing the need to infer their identity through linguistic, cognitive, and socio-cognitive processing; cognitive work which, in real-life word learning contexts, may divert processing resources from the process of word learning itself (Just & Carpenter, 1992). This is only one of many differences between experimental conditions and real-world word learning which may account for the differences in performance between contexts.

Such an explanation would align with the recent explanatory framework for LI posited by Ullman and Pierpont (2005): the Procedural Deficit Hypothesis (PDH). The PDH posits that children with LI have deficits in the procedural memory system (involved in implicit learning, storage, and use of knowledge) but that declarative memory (conscious, episodic knowledge), which is thought to underlie lexical knowledge, remains intact. Working memory (WM) impairments are not thought to be causal to LI but, rather, to be probabilistically associated with LI due to the anatomical proximity of the brain structures upon which both Procedural and WM processes depend. Word learning difficulties in LI, from this theoretical standpoint, are posited to result from the effects of the child's existing language knowledge on the process of word learning, which may be exacerbated, in some children, with co-morbid working memory difficulties. Further work is required to explicitly test this hypothesis, by examining word learning in LI in tasks with varying working memory demands, but such an explanation would certainly align with the findings of the current study.

Furthermore, it should be noted that the effect sizes in this study are very small and so the influence which PP and ND exert on the process of triggering should not be overstated; they are two variables amongst many which can potentially explain the variance in children's word learning abilities (Golinkoff *et al*., 2000). It is possible that the poor word learning in children with LI is, in fact, a multifactorial phenomenon whose causes may differ between children and over development (Hirsh-Pasek *et al*., 2000).

### The interactive effects of PP and ND

This study demonstrated that, overall, the optimal PP/ND characteristics for triggering word learning were either Low PP/Low ND or High PP/High ND, therefore aligning with the findings of Hoover *et al*. (2010) and extending their findings to demonstrate that this effect is also present for children with LI. As Hoover and colleagues point out, this ‘convergence advantage’ must change across development as the interactions between PP and ND found in preschool children are not found in adult word learning (Storkel *et al*., 2006). However, developmental change in this aspect of processing was not evident in their study (Hoover *et al*., 2010). The current study, however, provides empirical evidence for developmental change in this bias through the identification of a significant ND × PP × Vocabulary interaction. When unpacked, through post-hoc tests, this result was found to represent the fact that words with ‘divergent’ PP/ND characteristics (i.e., High PP/Low ND and Low PP/High ND) were harder to learn than those with ‘convergent’ characteristics (i.e., High PP/High ND and Low PP/Low ND), and that words with ‘divergent’ PP/ND characteristics were positively correlated with vocabulary knowledge, whereas words with ‘convergent’ characteristics were not. Therefore, as children's vocabulary knowledge grew, the disadvantage for words with ‘divergent’ ND/PP decreased. This relationship with vocabulary growth lends support to Hoover *et al*.'s suggestion that children's phonological and lexical representations may be more dependent on one another than in adults, but that this interdependence reduces as more robust lexical and phonological representations are specified: a process thought to be driven by vocabulary growth (Beckman & Edwards, 2000; Edwards *et al*., 2011; Garlock *et al*., 2001; Gupta & Tisdale, 2009; Pierrehumbert, 2003; Walley *et al*., 2003; Werker & Curtin, 2005).

Why would less well specified lexical and/or phonological representations mean greater interactivity between phonological and lexical levels of processing? The nature of interactivity in adult lexical processing continues to be debated, with models varying with respect to the nature of feedback, competition, bottom-up and lateral inhibition, and the nature of sublexical representations and processes (Frauenfelder, Scholten & Content, 2001; Magnuson, McMurray, Tanenhaus & Aslin, 2003; McClelland, Mirman & Holt, 2006; Norris, McQueen & Cutler, 2000). It is possible that, in an immature processing system, wherein sublexical/phonological representations are not yet fully developed, all processing would therefore involve lexical level representations to some degree. The independent lexical and phonological processing seen in adults could only occur once robust phoneme level representations have emerged which are separate from the lexical items in which they are attested (Edwards *et al*., 2011; Munson, Kurtz & Windsor, 2005; Pierrehumbert, 2003). As the emergence of phoneme representations is thought to be an incremental one, driven by vocabulary growth (Beckman & Edwards, 2000; Edwards *et al*., 2011; Garlock *et al*., 2001; Gupta & Tisdale, 2009; Pierrehumbert, 2003; Walley *et al*., 2003; Werker & Curtin, 2005), the correlation identified here between improving word learning ability for words with diverging ND/PP characteristics with vocabulary growth would support such an explanation.

### Conclusion and future recommendations

The influence of PP/ND characteristics on triggering word learning in children with LI does not differ from TD children when compared with respect to vocabulary knowledge. These results therefore do not support the hypothesis that phonological processing is a core deficit in LI (Bishop, 2000; Bowey, 2006; Chiat, 2001; Maillart *et al*., 2004; Mainela-Arnold & Evans, 2005; Seiger-Gardner & Brooks, 2008), although such a deficit could perhaps be present in other stages of word learning. The search for the source of the word learning difficulties found in this group of children must, therefore, continue.

If we are to fully understand the nature of lexical and phonological processes involved in word learning in TD children and those with LI, further research is needed which fully crosses ND and PP, explores triggering, configuration, and engagement, and which considers the issue of developmental change. However, the results from the current study and those of Gray and colleagues (Gray & Brinkley, 2011; Gray *et al*., 2012) and Kan and Windsor (2010), together with the Procedural Deficit Hypothesis of LI (Ullman & Pierpont, 2005), would suggest that a broader research agenda may be needed, considering the cognitive as well as the linguistic demands of word learning. Future research to determine the source of the word learning difficulties in LI should explore the many demands of word learning in the ‘real world’, such as the influence of differential demands on processing capacity, social cognition, statistical learning, attention, and memory processes, together with the complex nature of their interactions and the process of developmental change (Golinkoff *et al*., 2000; Hirsh-Pasek *et al*., 2000).

## Footnotes

[1] LI is used here to refer to children traditionally diagnosed as having a Specific Language Impairment. The term ‘LI’ is preferred as the concept of ‘residual normality’ implied by the use of the term ‘specific’ is questioned by the authors on both empirical and theoretical grounds.

[2] Error data were analyzed but did not yield informative results and so are not presented here. These results are available from the authors.

## APPENDIX: NUMBERS OF PARTICIPANTS BY AGE GROUP

**Table:**

Age range (months)	N	
	TD	LI
37–47	12	5
48–59	20	3
60–72	6	1
73–84	0	3
